# Digitally Guided Soft-Tissue Recontouring Using a 3D-Printed Surgical Stent for Aesthetic Rehabilitation of a Patient With Moderate Dental Fluorosis: A Case Report

**DOI:** 10.7759/cureus.108493

**Published:** 2026-05-08

**Authors:** Chanda Yadav, Shruti Tandon, Arundeep K Lamba, Farrukh Faraz, Akshita Mehta

**Affiliations:** 1 Periodontics, Maulana Azad Institute of Dental Sciences, New Delhi, IND; 2 Prosthodontics, Maulana Azad Institute of Dental Sciences, New Delhi, IND

**Keywords:** 3d-printed surgical guide, dental fluorosis, digital smile design, esthetic rehabilitation, gingivectomy

## Abstract

Generalized dental fluorosis presents aesthetic challenges due to enamel discoloration, surface pitting, and irregularities. Management often requires correction of both gingival architecture and intrinsic tooth defects. This report describes the use of a digital workflow in a patient with generalized dental fluorosis that includes Digital Smile Design (DSD Company, Madrid, Spain), Exocad dental computer-aided design (CAD) software (Exocad GmbH, Darmstadt, Germany) for mock-up, accurate evaluation of gingival contour, biological width, veneer planning, and a three-dimensional (3D)-printed periodontally guided surgical template for gingivectomy followed by lithium disilicate veneers. A 26-year-old female patient presented with a chief complaint of asymmetrical gingival display and brown discoloration of teeth. Clinical examination revealed generalized dental fluorosis with irregular gingival contours extending from tooth 14 to tooth 24, with probing depths within normal limits (2-3 mm). Extraoral and intraoral photos were used to create a digital smile design. The suggested result was then imported into Exocad software to create a diagnostic mock-up. A 3D-printed surgical guide was fabricated to perform a gingivectomy in accordance with the intended gingival contours. Following healing, minimally invasive preparations were carried out, and the digital design was used to fabricate lithium disilicate veneers. At the 12-month follow-up, a stable and harmonious gingival margin was seen with excellent veneer adaptation without any complications. This association of DSD, CAD-based digital mock-up, 3D printing of surgical guides, and lithium disilicate veneers made a predictable, minimally invasive, and aesthetic rehabilitation in a patient with generalised dental fluorosis.

## Introduction

Since 2007, Digital Smile Design (DSD) (DSD Company, Madrid, Spain), introduced by Coachman, has been widely utilized in aesthetic dentistry through the integration of digital workflows, which enable practitioners to perform smile design in a more precise manner [1].

Dental fluorosis is a qualitative defect of enamel resulting from an increase in fluoride concentration within the micro-environment of the ameloblasts during enamel formation [2]. Dental fluorosis has been classified by Dean into six categories based on severity: normal, questionable, very mild, mild, moderate, and severe, ranging from mild changes in enamel translucency to marked pitting and brown discoloration [3].

The use of DSD offers a dynamic and patient-centered method for diagnosis and treatment planning by visualizing the results before treatment. The use of intraoral scanning and computer-aided design/manufacturing technology helps in the fabrication of three-dimensional (3D)-printed surgical guides for gingivectomy and crown lengthening surgeries, which gives accurate, efficient, and safe results from a biological perspective [4-6]

Recent publications highlight that digitally controlled periodontal treatments achieve better results than the freehand technique, with better control of gingival zeniths, symmetry, and preservation of supracrestal attachment [7-9]. Application of provisional mock-ups, which are based on digital planning, improves interdisciplinary communication and patient acceptance. Lithium disilicate veneers have provided an ideal restorative option in the case of fluorosis due to their high mechanical strength, optical characteristics, and conservative preparation after soft tissue optimization as compared to feldspathic porcelain [10,11].

This case report presents the application of DSD and 3D-printed surgical guides to perform gingivectomy in a patient with generalized dental fluorosis, followed by veneers for definitive rehabilitation.

## Case presentation

A 26-year-old female patient reported to the Department of Periodontics with a chief complaint of uneven display of gums with brown discolouration of teeth, which made her self-conscious while smiling. Medical history revealed noncontributory findings. There were no systemic contraindications to undergo dental treatment. Family history revealed that the patient’s family members had similar tooth discolourations. 

**Figure 1:**
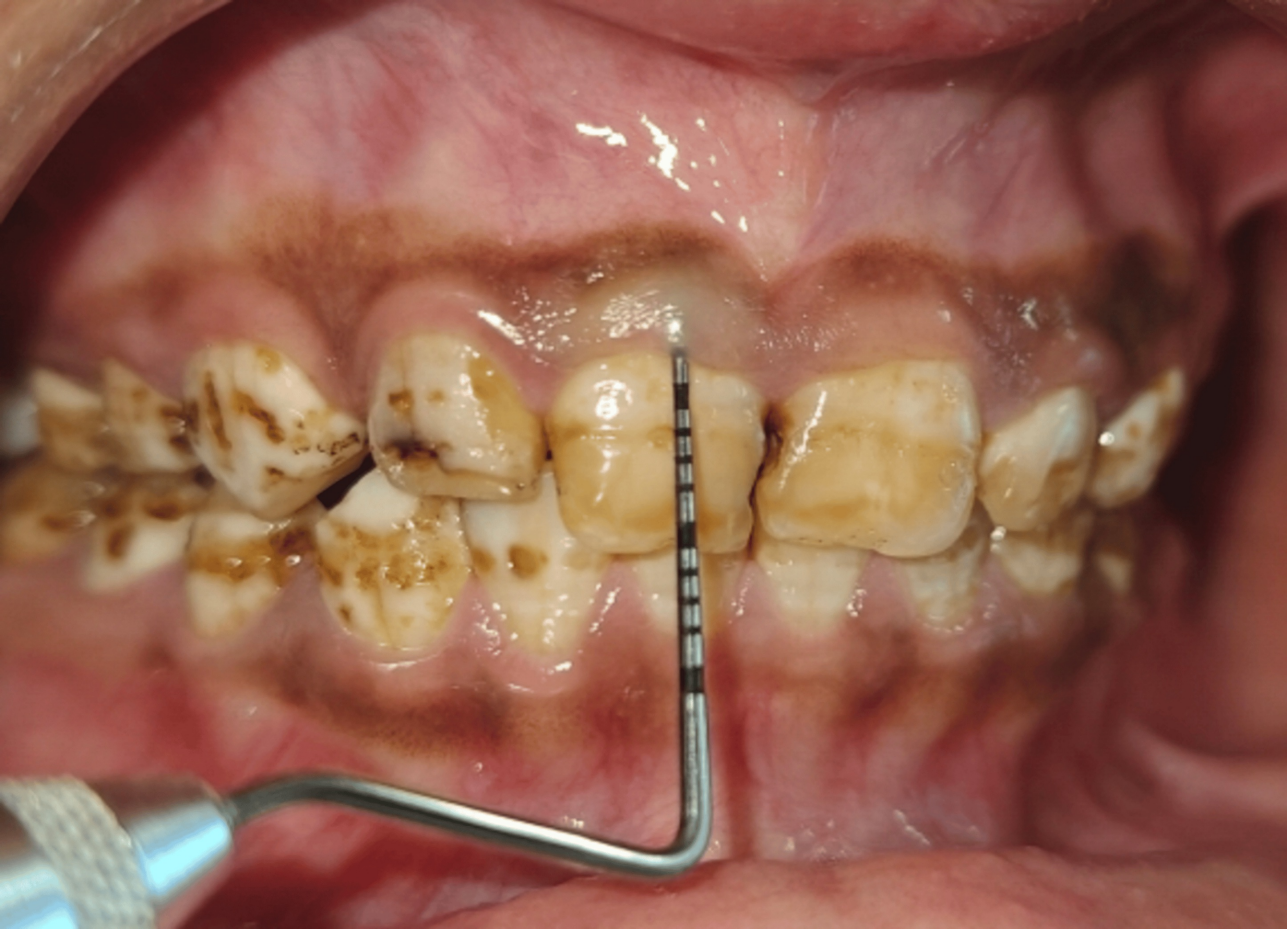
Preoperative view showing normal gingival sulcus

**Figure 2:**
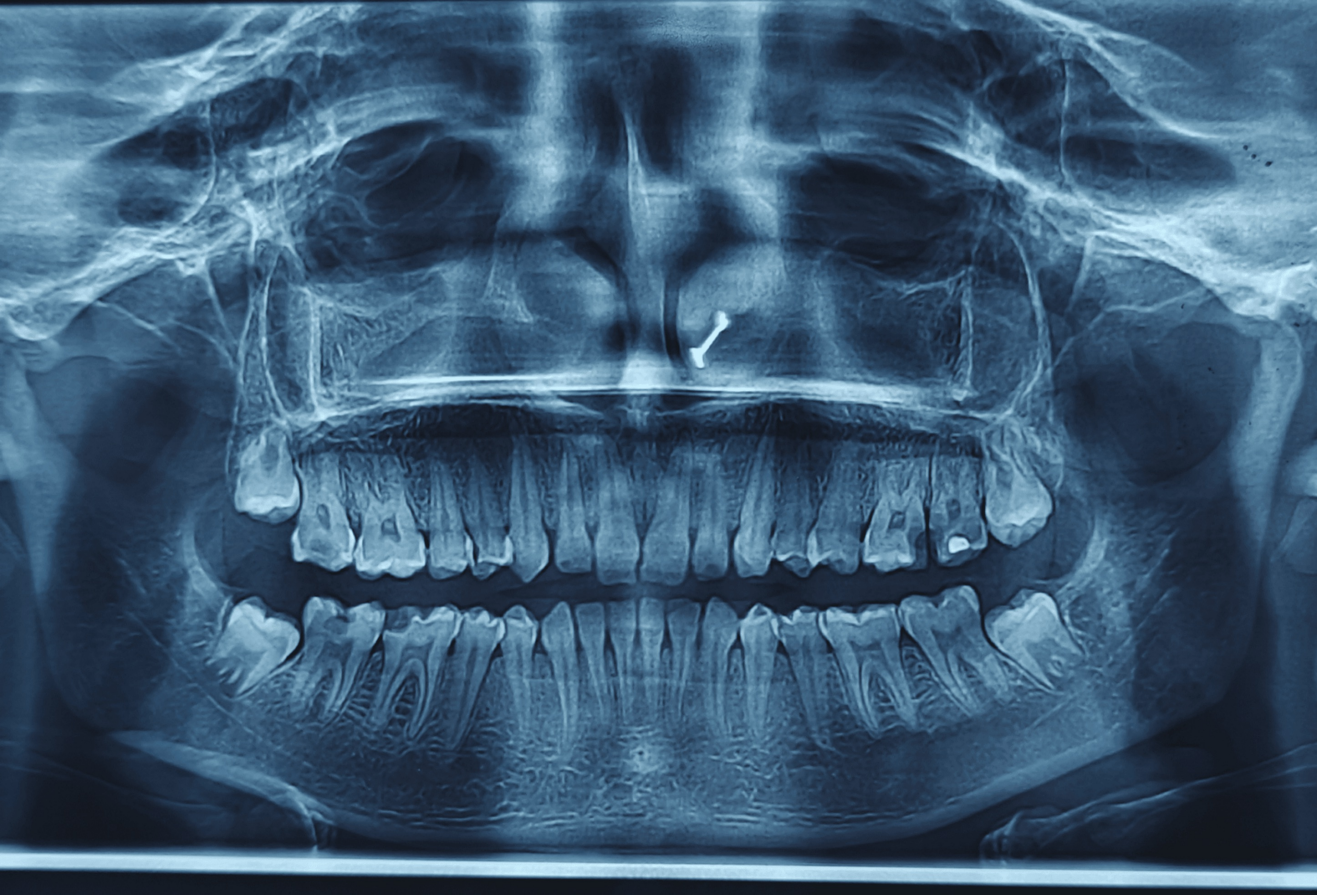
Orthopantomograph (OPG) revealing normal bone level

On clinical examination, there was a gingival display of 2 mm on smiling, as well as disharmonies in the gingival contours. The probing depths were found to be within the normal limits; it was 2-3 mm in the region extending from tooth 14 to tooth 24 (Figure 1). There were no signs of attachment loss or inflammation. Bone sounding was done under local anaesthesia to determine the supracrestal tissue attachment. The tissue attachment was found to be 4 mm. This was adequate space for recontouring the tissue without any resection of the bone (Figure 2). 

**Figure 3:**
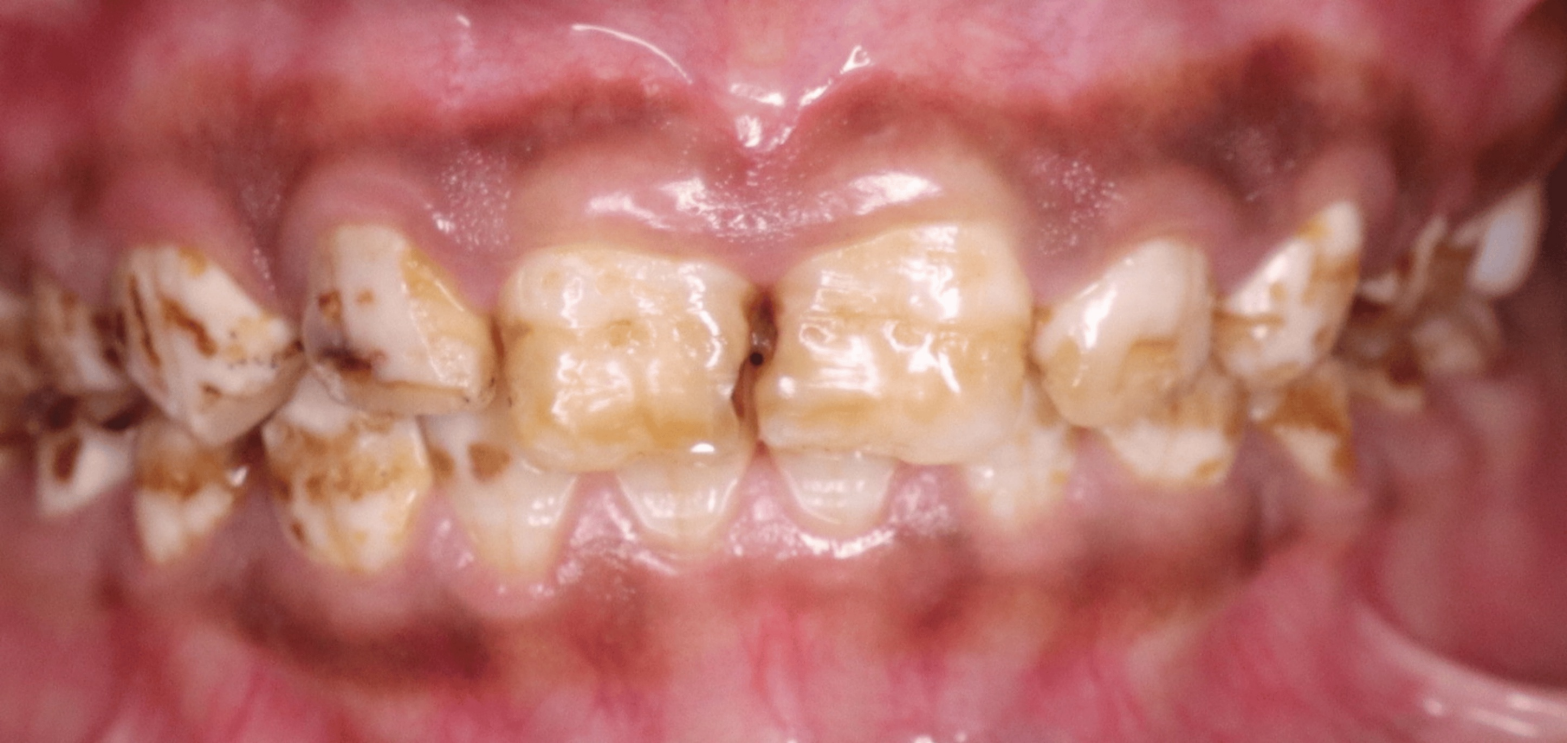
Preoperative smile showing generalized dental fluorosis and asymmetric gingival contour

The patient had generalised dental fluorosis involving both the maxillary and mandibular teeth. The maxillary anterior teeth had signs of brown mottling, loss of translucency of the enamel, as well as mild surface pitting (Figure 3). The dental fluorosis was classified as a moderate case of fluorosis based on the results of the Dean Index for fluorosis [3]. Occlusal analysis revealed that the overjet and overbite were within normal limits. There were no signs of parafunction. 

Digital planning

**Figure 4:**
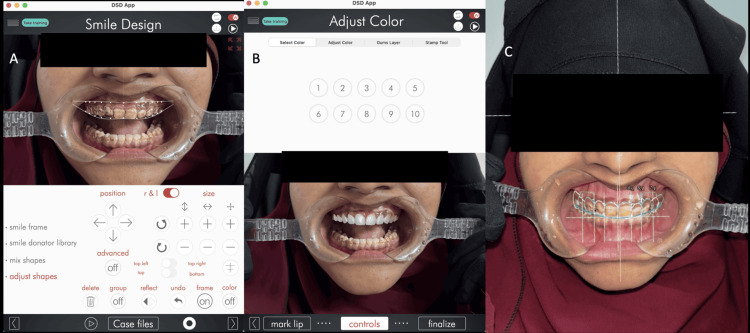
Using Digital Smile Design software in aesthetic analysis and treatment planning (A) The preoperative frontal smile photograph with retractors was used as the baseline for analysis and generation of the smile design framework. (B) This was followed by a virtual simulation of the proposed tooth morphology and shade selection using Digital Smile Design software (DSD Company, Madrid, Spain). (C) Subsequently, digital reference lines and proportional grids were superimposed to evaluate the dental midline, incisal plane orientation, tooth proportions, and overall smile symmetry.

Digital planning was initiated by capturing standardised extraoral and intraoral photographs with the patient in natural head position and full smile. Photographs were imported into the DSD software environment to establish horizontal reference lines (interpupillary and commissural lines) and vertical midline references. Proportions of the maxillary anterior teeth were digitally analysed according to established smile arch harmony and aesthetic principles (Figure 4). Tooth proportions were evaluated using the golden proportion concept described by Levin (1978) [12], assessing the relative mesiodistal widths of the maxillary anterior teeth as viewed from the frontal perspective.

**Figure 5:**
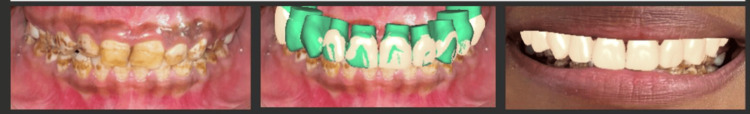
Two-dimensional visualization of diagnostic mock-up superimposed on intraoral frontal view

**Figure 6:**
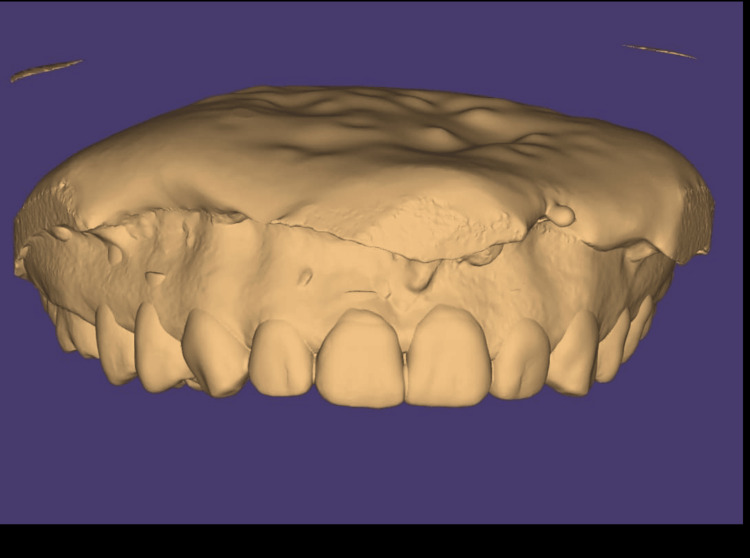
Three-dimensional model of diagnostic wax-up

**Figure 7:**
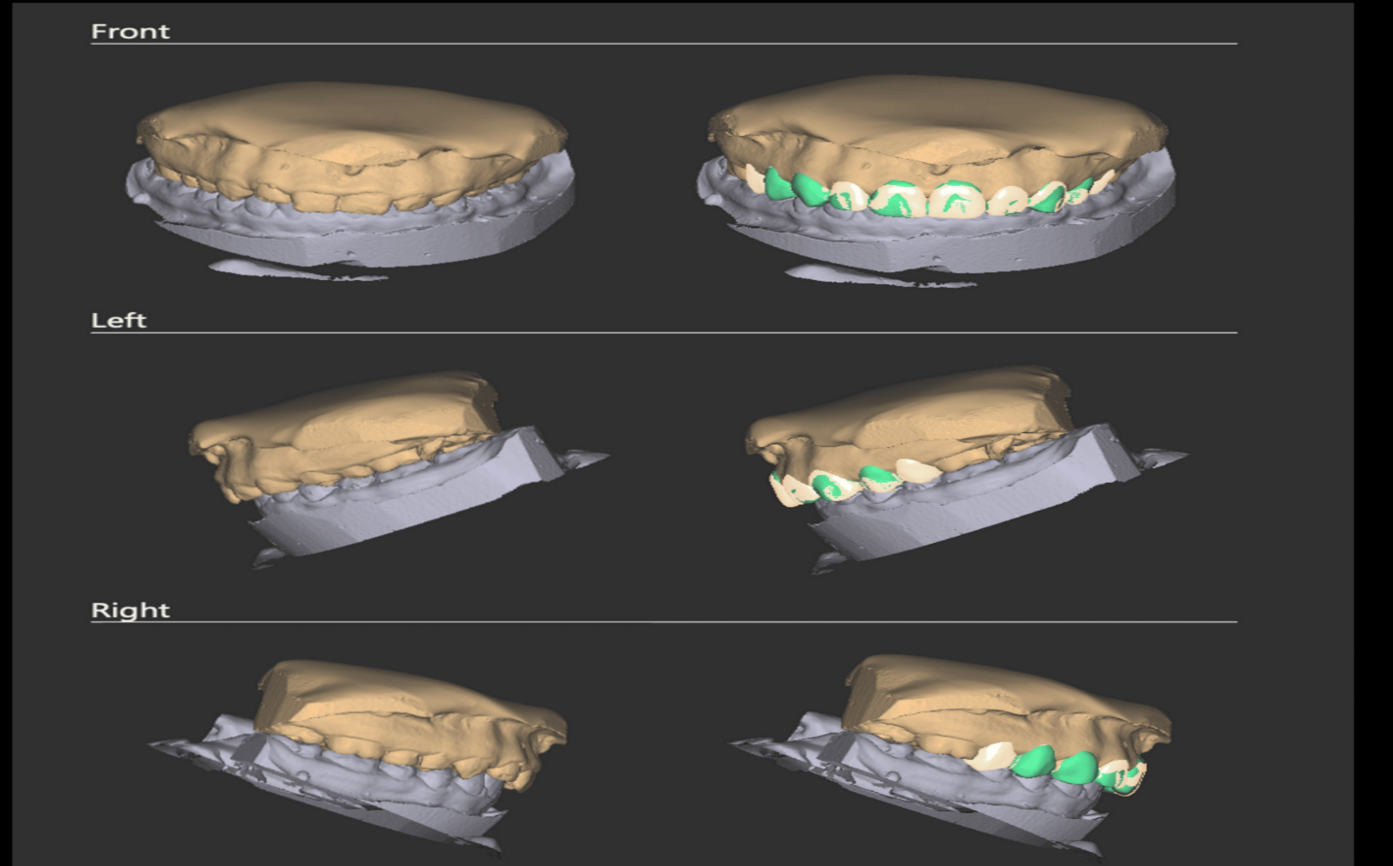
Exocad dental CAD* digital workflow illustrating virtual diagnostic wax-up and planned gingival recontouring for the maxillary anterior teeth *Exocad GmbH, Darmstadt, Germany CAD: computer-aided design

**Figure 8:**
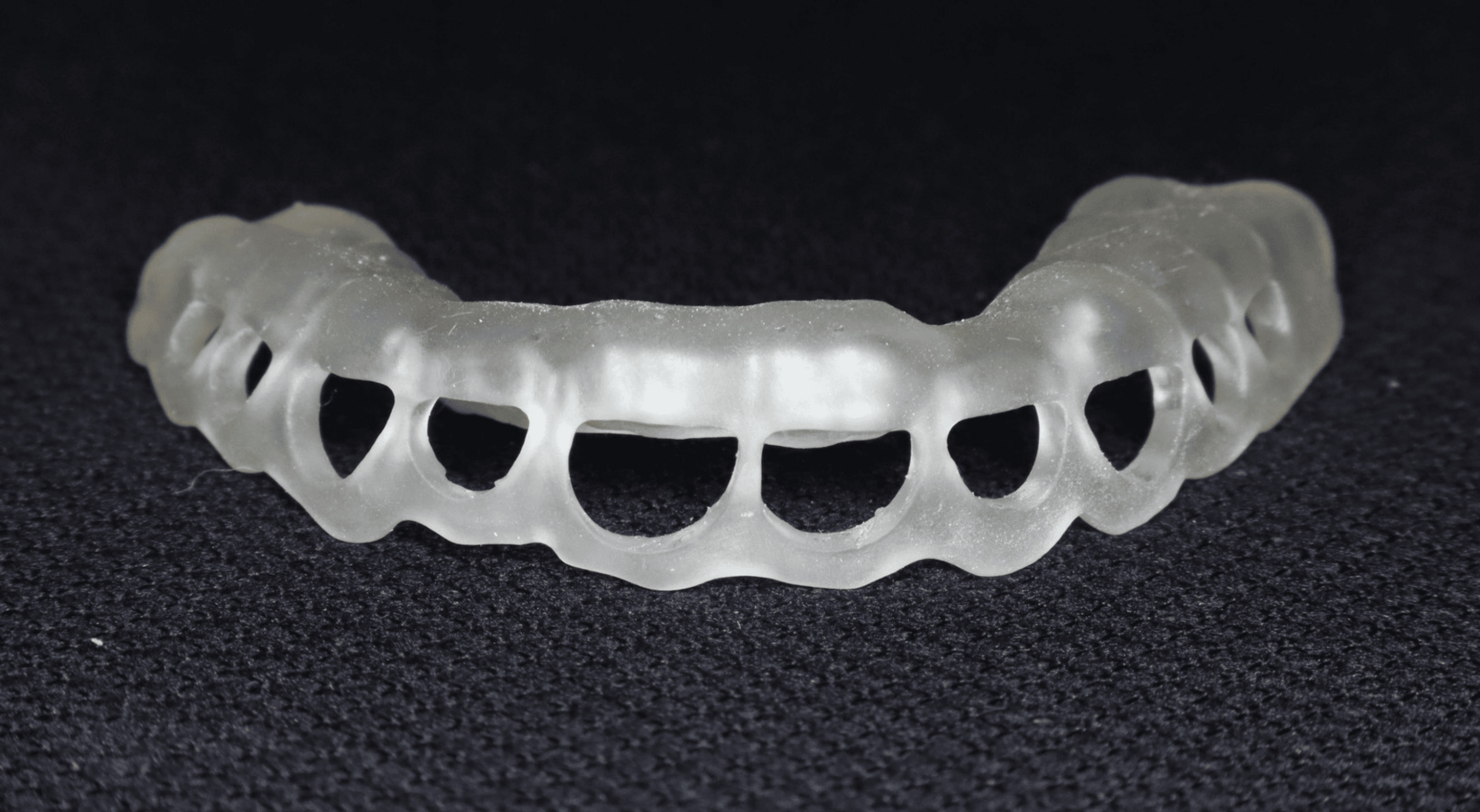
Three-dimensional-printed surgical guide fabricated using stereolithography (SLA) technology with BioMed Clear* *Formlabs,  Somerville, Massachusetts, United States

While the golden proportion provided a useful guideline, individual variations in tooth proportions were observed and considered during aesthetic evaluation. The approved aesthetic analysis was then exported to Exocad Dental CAD software (Exocad GmbH, Darmstadt, Germany), where it was superimposed on the patient’s STL (Standard Tessellation Language) files from the intraoral scan. Exocad was used to create a digital diagnostic wax-up that simulated final tooth proportions and gingival zenith positions. This enabled a virtual mock-up to be visualised in both two-dimensional (2D) (photographic overlay) and 3D (STL model) formats, as shown in Figure 5 and Figure 6, respectively. Using this mock-up, a surgical guide was developed that enabled accurate gingivectomy in accordance with the gingival contours. The guide was created using a 3D printing method known as stereolithography (SLA) 3D-printing technology and BioMed Clear resin (Formlabs, Somerville, Massachusetts, United States) (Figures 7, 8).

Surgical phase

**Figure 9:**
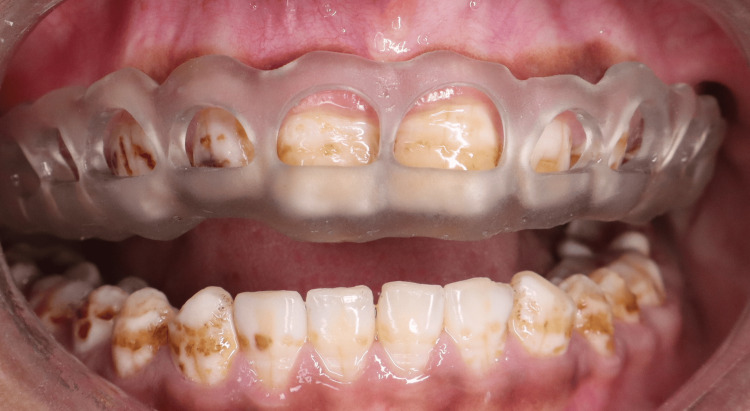
Intraoral positioning of the surgical guide prior to gingivectomy to verify fit and stability.

**Figure 10:**
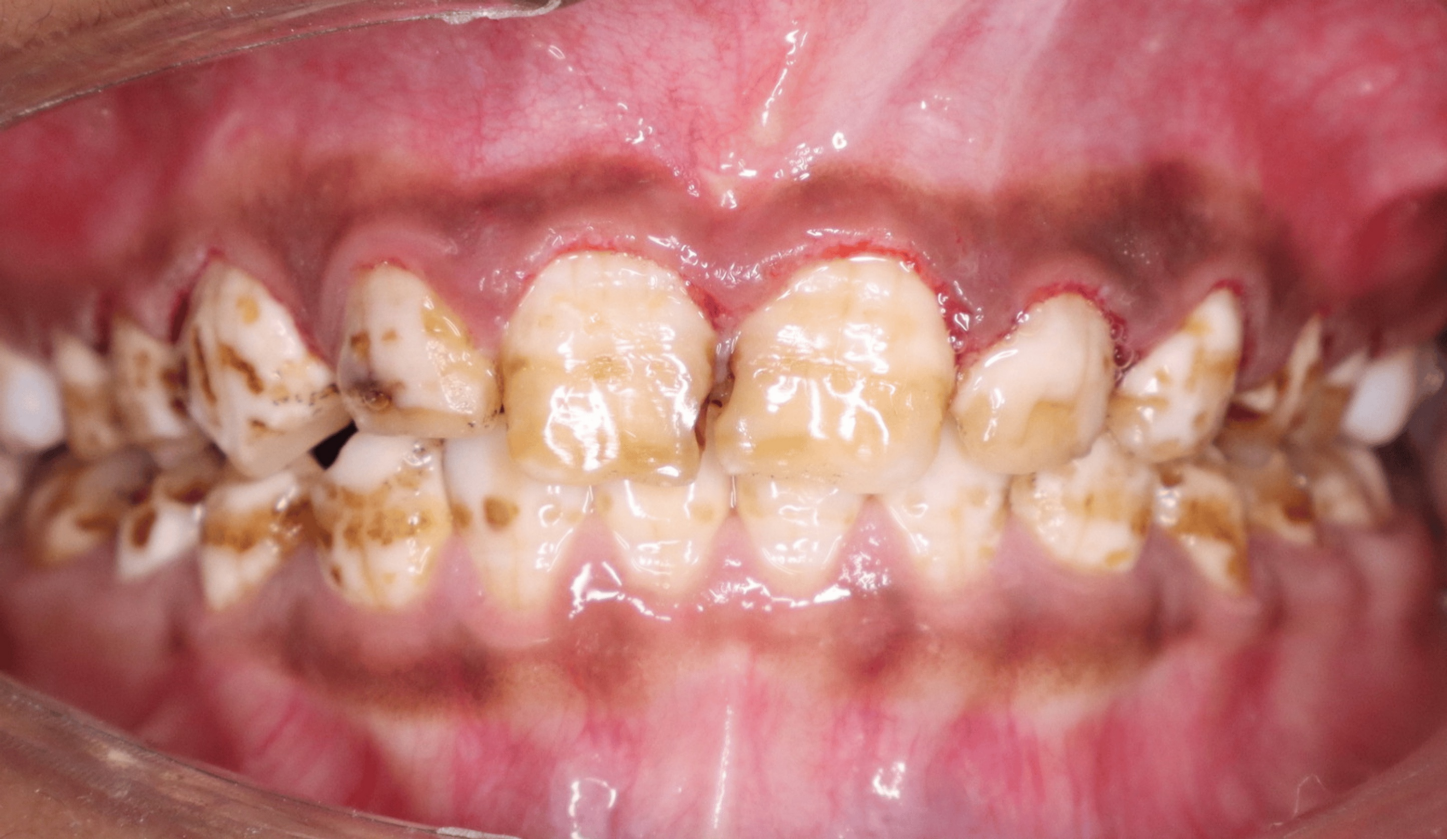
External bevel gingivectomy performed using the surgical guide to achieve the planned gingival contour

**Figure 11:**
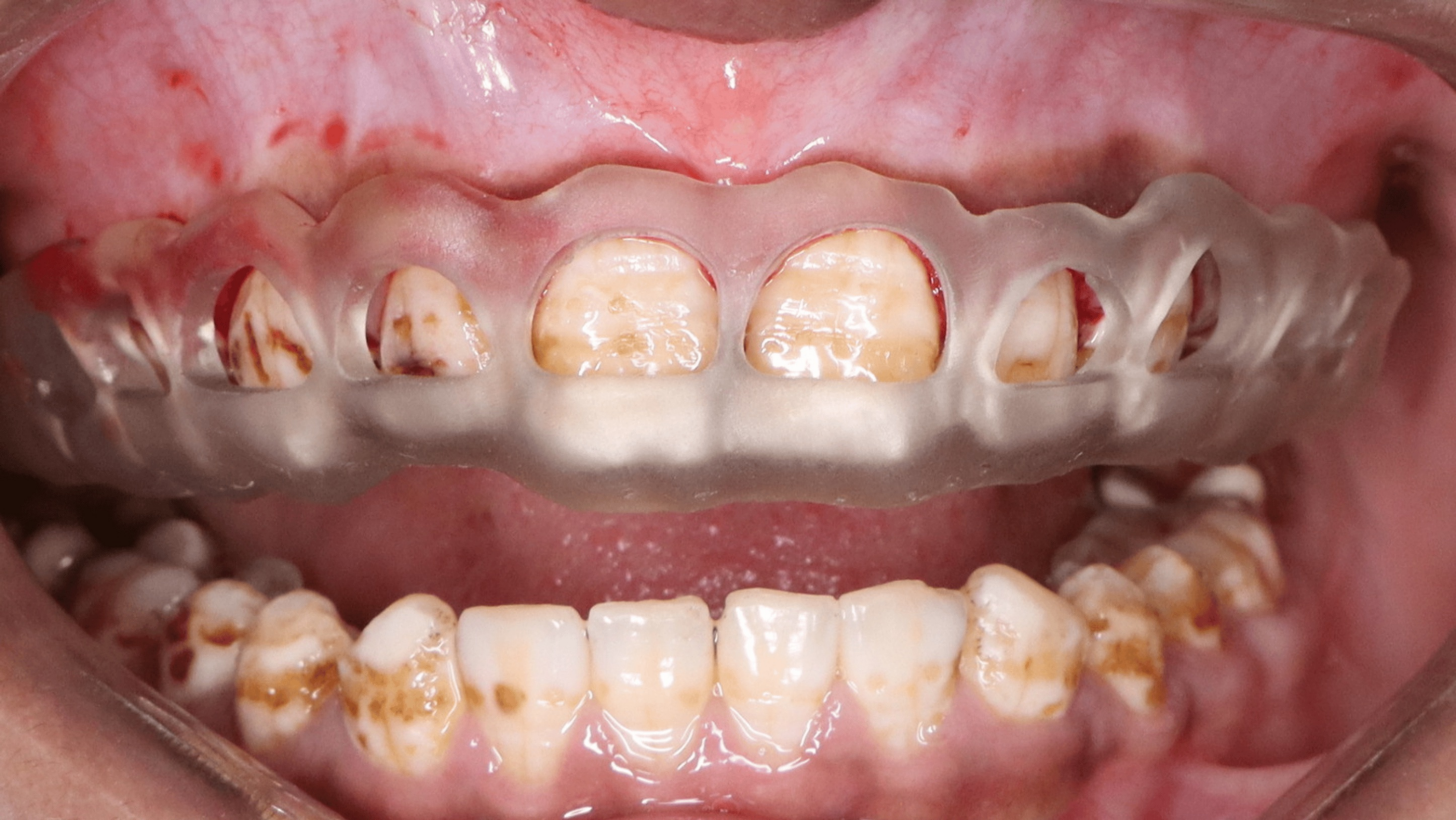
Post-surgical view showing recontoured gingival margins after guided gingivectomy.

Gingivectomy was conducted utilising a tailored 3D-printed surgical guide. The surgical site was anaesthetised with local anaesthesia comprising 2% lignocaine and 1:80,000 adrenaline. The 3D-printed guide was positioned intraorally, and its fit was verified clinically by checking complete seating, stability, and adaptation to the dental structures prior to use (Figure 9). The gingival tissue designated for removal was outlined through the windows of the template. An external bevel gingivectomy was performed with a #15C blade, adhering to the markings indicated by the 3D-printed guide (Figures 10, 11). The excised gingival tissue was removed, and hemostasis was achieved through the application of local pressure. The site was subsequently irrigated with saline solution, followed by the application of a periodontal dressing. Postoperative instructions were provided, including prescription of analgesics (Ibuprofen 400 mg as needed for pain) and 0.12% chlorhexidine mouthwash twice daily for two weeks. The healing process was monitored during follow-up appointments at one week and four weeks, with a subsequent 12-month evaluation to assess long-term gingival stability.

Restorative phase

**Figure 12:**
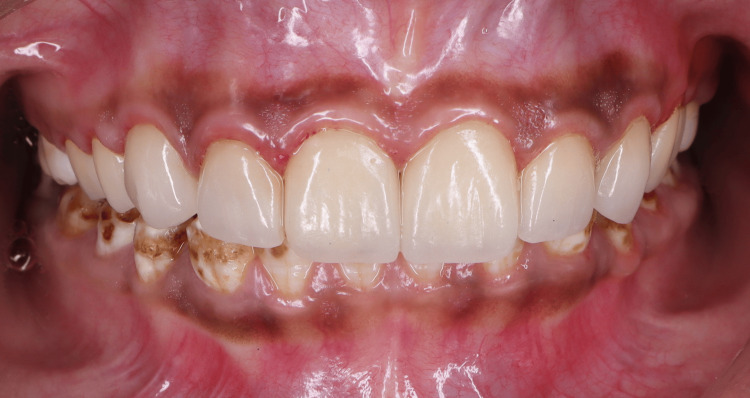
Final lithium disilicate veneer restorations after cementation.

Four weeks after soft tissue stabilization, an incisal overlap design of veneers was performed, guided by the Exocad mock-up with minimal preparation. Lithium disilicate veneers were fabricated (IPS e.max Press; Ivoclar Vivadent AG, Schaan, Liechtenstein), replicating a digitally designed mock-up. The internal surfaces of the lithium disilicate veneers were etched with 5% hydrofluoric acid (Porcelain Etch; Ultradent Products, Inc., South Jordan, Utah, United States) for 20 seconds and then treated with a silane coupling agent (Silane; Ultradent Products, Inc.) for 60 seconds before cementation. Following etching and silanisation, veneers were bonded with dual-cure resin cement (RelyX™ Ultimate Adhesive Resin; 3M Company, Maplewood, Minnesota, United States) following the manufacturer’s instructions (Figure 12).

Follow-up

The patient was reviewed after one week for initial healing assessment, and a clinically appreciable increase of 1 mm in clinical crown height was observed in anterior teeth following gingivectomy. There was a follow-up at four weeks for evaluation of soft tissue stabilisation, and periodically thereafter. At the long-term follow-up at 12 months, to assess gingival stability and overall periodontal health, the gingival margins were stable. Gingival health was stable with the absence of gingival recession, no signs of inflammation, and no bleeding on probing. Periodontal assessment revealed that probing depths (1-2 mm) were normal, and veneers showed excellent aesthetic integration with no complications. 

## Discussion

The digital workflow facilitates well-defined transfer of intended gingival contours into the clinical setting. The 3D-printed surgical guide enabled clinically acceptable accuracy in gingival contour transfer, allowing gingivectomy without the need for osseous recontouring, aligning with earlier studies on digitally planned soft tissue surgeries that guarantee predictability and symmetry [4-6]. Lithium disilicate veneers preparation using Exocad mock-up produced successful masking of fluorosis-related discolorations and surface defects, providing high translucency and aesthetic properties. This is similar to the use of lithium disilicate ceramics as veneers for managing intrinsic enamel defects, as deeper fluorosis stains may also be considered intrinsic in nature, as previously reported [9-11].

The integration of the restorations with the newly harmonized gingival architecture resulted in a pleasing and balanced smile. The patient experienced a marked improvement in aesthetics and confidence. The patient was satisfied with both the process and the results. At the 12-month follow-up, the gingival margins were stable and healthy. There was no recession and no bleeding on probing. The veneers did not show evidence of debonding, discoloration, or secondary pathology. Such results suggest that digitally planned aesthetic rehabilitations can be stable in the long term [7,8].

To have optimal results in aesthetic dentistry, it is crucial to consider a variety of factors such as the morphology of the teeth, shade, the structure of the gums, the lip process, and the symmetry of the smile [11,13]. A multidisciplinary approach, including periodontal and restorative procedures, is therefore likely to produce the best aesthetic results. Treatment of patients with generalized dental fluorosis becomes especially difficult because of intrinsic enamel discoloration, pitting of the surface, and loss of enamel translucency, so both soft-tissue correction and restorative rehabilitation are required.

Traditional gingivectomy techniques are mainly operator-dependent and highly rely on clinical judgment to establish the optimal gingival margin. These freehand techniques can result in asymmetrical gingival contours and non-uniform gingival zeniths, and have a possible risk of violating supracrestal tissue attachment unless the soft tissue removal is controlled properly [14]. Moreover, when treatment is planned by conventional diagnostic methods, it becomes challenging to visualize the end result of aesthetics during the surgery [4]. Although widely used, diagnostic wax-ups can occasionally exaggerate the optimal gingival contour, which could result in unwarranted removal of the soft tissues and root exposure [15]. These constraints highlight the need for more predictable, reproducible, and less invasive methods of soft-tissue recontouring.

Digital planning has emerged as a reliable substitute to traditional methods since it creates a better visualization and treatment predictability. DSD integration with CAD software enables clinicians to examine the proportions of the face, size of the teeth, location of the gingival zenith, and dynamics of a smile prior to the beginning of treatment [16]. This digital workflow helps clinicians make better diagnoses and simulate the proposed aesthetic outcome more accurately. Thus, it improves communication between the patient, laboratory technician, and clinician [9]. Digital planning software enables the creation of a patient-specific guide that outlines the exact contour and height of soft tissue resection [17].

 The 3D-printed surgical stent defines the planned gingival margin and directs the surgical blade, which minimizes unwarranted movements during the gingivectomy procedure. The stent allows soft-tissue recontouring according to the pre-planned digital design by providing a fixed reference point and reducing the risk of excessive or insufficient gingival resection. This increases the precision of the surgery and results in better aesthetic and functional outcomes. It makes intraoperative decisions easier since all the diagnostic and aesthetic planning is transferred directly to the surgical field. This reduces uncertainty during surgery, minimizes subjectivity, and improves the reproducibility and standardization of the procedure.

This case highlights the role of digital workflows in improving the predictability of periodontal and restorative procedures, and shows that the use of AI to assist smile design, integration of dynamic smile analysis, and chairside CAD/computer-aided manufacturing (CAM) veneers may streamline this workflow, making digital aesthetic rehabilitation more efficient and accessible in the future. However, this report represents a single clinical case with a short-term follow-up period, and additional research is needed to assess the long-term stability of digitally guided gingival recontouring. 

## Conclusions

The integration of DSD, Exocad mock-ups, and 3D-printed guides facilitates predictable aesthetic rehabilitation of generalized fluorosis. Lithium disilicate veneers successfully masked discolouration and restored natural esthetics. Within the limitations of this report, it is seen that digital workflows may contribute to more consistent aesthetic rehabilitation outcomes.
